# Buffalo Medical and Surgical Association

**Published:** 1890-05

**Authors:** W. H. Bergtold

**Affiliations:** Secretary


### BUFFALO MEDICAL AND SURGICAL ASSOCIATION.

Reported by W. H. BERGTOLD, M. D., *Secretary*.

REGULAR monthly meeting, held Tuesday evening, March 4th, at 8.30 o'clock, in the Library of the Polytechnic Institute, No. 9 West Mohawk street.

Drs. B. N. STRONG and E. N. Pfohl were proposed for membership.

Dr. A. A. JONES was invited to participate in the meetings of the Association until such time when he could perfect his membership.

Dr. HERMAN E. HAYD then read a paper on

### ELECTRICITY IN GYNECOLOGICAL PRACTICE.

No matter in what department of medicine or surgery where faithful enquiry is made, there comes universally good reports from the application of electricity for the relief and cure of certain diseased conditions; yet there is no remedial agent which has ever been subjected to such unkind criticism, or to such varied and unscientific uses, as this powerful agent has. Taken from the hands of charlatans and street venders, ignorant and unscrupulous persons, it now has a recognized place in scientific medicine; and its therapeutical properties are acknowledged by the best men in the profession. Of the various forms of electricity that are used, I wish to invite attention to the practical application of the faradic and galvanic currents in the treatment of diseases of the uterus and its appendages. In a general way, I may say that I have found the faradic current especially useful in overcoming the more recent forms of subinvolution, accompanied by relaxed vaginæ, and more or less prolapsed uteri; as well as in relieving and positively curing constipation due to, or consequent upon, and accompanying all forms of uterine disease—in a word, in benefiting those conditions, where muscular tonicity and contractility are deficient. On the other hand the galvanic current, with its proper polar application, has given the most encouraging and even surprising results in the treatment of all kinds of chronic inflammations, congestions, and hypertrophies, existing either in the mucous membrane or substance of the body, or neck of the womb; malpositions and displacements; inflammatory adhesions about the uterus and its appendages; tubal and ovarian irritations, and organic diseases of them.

In the practical application of galvanism we have two poles: one the inactive, which is usually applied by means of a large pad externally, either near or upon the diseased territory; and the active or internal pole, which applies the electric force directly upon and into the diseased tissues. These poles are called positive and negative, and differ widely not only in their chemical but therapeutical properties.

The positive generates oxygen and is acid in reaction, or better, liberates or generates acids from the tissues; while the negative gives off hydrogen, and is alkaline in reaction. Consequently, they differ not only in their topical action, but in what is called their polar action, or their physiological effect upon the tissues. The positive pole is styptic, caustic, hemostatic. It is also sedative, anti-congestive and depletive. It lessens the supply of blood to the part, by contracting the blood-vessels, and hence cuts off excessive nutrition, and increased vascularity; and, furthermore, brings on increased muscular contractility. Hence, it is indicated when there is a tendency to hemorrhage, when there is active discharge together with pain and tenderness, and where the tissues are swollen and soft, and in a condition of more or less subacute inflammation or congestion.

On the other hand the negative pole increases the circulation in the part, and in that way promotes absorption.

It is stimulating, absorptive, electrolytic, denutritive, and also caustic in its local effects; and consequently has its place for the more chronic inflammations, with their accompanying exudations and hypertrophies; yet experience will teach us that we can't be too arbitrary in the selection of our poles; nor must we always expect these definite results, as I have seen some recent cases of subinvolution, accompanied by profuse, irritating, more or less bloody, offensive discharges rapidly improve under negative galvanism. The explanation which I offer is that the mucous membrane was specially involved, and the caustic effect of the negative pole disorganizes and disintegrates the diseased tissues, and causes them to be rapidly exfoliated.

Let me now call your attention to the electrodes which I have been in the habit of using. I prefer for the external pad one of clay, in fact a modification of Apostoli's, made by Goelet, of New York. It possesses a great many advantages, is so easily applied, and can be kept warm; consequently, does away with the objectionable features associated with the use of cold, wet towels, over the wire gauze electrodes. In an excellent article by Goelet, in the *Medical News*, June 22, 1889, he describes at length the way these pads are made.

When positive galvanism is used, the electrode must be made of some incorrodible material; consequently, platinum is preferred. I have also used with satisfaction, a composition material suggested by Goelet. If any of the other baser metals be used, it will be found that the tissues stick to the electrode; consequently, it will be with difficulty withdrawn, and will also be found that the tips are intensely corroded.

For negative galvanism any material may be used, and, as a rule, copper tips, nickle-plated, answers the purpose satisfactorily.

A milliampère meter is absolutely necessary for the proper and scientific administration of electricity; and by it alone can the requisite dosage be estimated and regulated.

A water rheostat is also a great convenience, since it prevents the sudden concussions and unpleasant sensations often produced when the current is intensified.

In giving a patient electricity, it is well to commence with a weak current, and gradually increase it. At first, the sêances should be short, say two or three minutes; and in the course of a week or ten days, much longer sittings and currents of much higher intensities are well borne. If there be much tenderness present, or when even a weak current of twenty to thirty milliampères causes prolonged pain and distress or discomfort after an intra-uterine application, it is well to first give vaginal galvanism, applied to the os uteri and around it with a clay electrode—Goelet's, I prefer. When two or three—or in any very bad cases six—treatments of this character are administered, it will be found that the womb will tolerate the electrode internally, and very shortly permit currents of fifty to seventy milliampères, and even one hundred, to be employed with benefit and absolute freedom and safety. The treatments may be given from two to three times a week. It is well for the patient to recline on a couch in an ante-room after the application has been given, to overcome the slight shock, the neuralgic pains in the legs and back, nausea, and sometimes dizziness and faintness which are complained of. Moreover, it is well to insert into the vagina an antiseptic tampon of absorbent cotton after the treatment has been given. It is grateful to the patient by giving the parts support, and also has a tendency to check any hemorrhage which might occur, as well as takes up the watery discharge occasioned by intra-uterine application. The plug may be removed in the evening, or on the following morning.

For the relief of obstructive dysmenorrhea, I think there is no agent so universally applicable, and I take from my case book the following as an example:

CASE I.—A. W., age 34, single.—For years have suffered terrible pains, when unwell, in fact, has often taken morphine for relief, and of late years fluid extract black hawthorn in large doses, often repeated. Is regular, but periods come every twenty-one days; and this has been her habit through life. Was operated upon seven years ago, and for a short time had relief, but apparently the operation has done her no permanent good. Suffer for twenty-four hours acutely in her back, and hips, and groins, until a show is noticed, and then considerably, during the whole period, which usually lasts three or four days.

Diagnosis: extreme corporeal anteflexion with pointed pin-holed os, —enlargement of walls of uterus; sound passed in, acutely bent, about one and three-quarter inches; endometrium very sensitive.

Patient was treated twice a week, the clay electrode applied over the abdomen, and a flexible platinum-pointed electrode inserted as far as possible into the uterus. The galvanic positive pole was used internally at first, and forty ma. current was given. The sittings lasted from five to fifteen minutes, and the intensity ranged from forty to seventy-five ma. The patient took three treatments a week, until a good deal of vesical irritability was complained of, when the seances were given less frequently. The first period came on as usual, and positively no pain whatever was complained of. The treatments were continued for two months longer, and at the last sitting the sound entered two and one-quarter inches. The interval between the second and third menstrual period was six weeks. Now nine months have passed by since electricity was first administered, and seven menstruations have occurred, without the slightest discomfort to the patient. She gained in flesh six pounds, and her general physical condition is much improved.

The treatments were discontinued, as I have said, on account of the vesical irritability complained of, together with the fact that the patient was completely relieved of the condition for which she sought medical advice. For the relief of this condition authorities differ. Some prefer the positive pole, claiming that the resulting eschar is more permanent, and, consequently, the opening more patulous. I have preferred the negative, since, in its application, the tissues being relaxed, the electrode enters with less difficulty, and, if desirable, can be passed further into the uterine cavity, and correct, or, at all events, benefit the malposition.

CASE II.—*Chronic subinvolution with enlargement. Sound enters three and three-quarter inches; tissues hard, and firm; walls of uterus thick; anteversion, endocervicitis, erosion of os, especially posteriorly; pelvic adhesions, especially to left side. Relaxed vagina, with some prolapsus uteri.*

Patient *æt.* 24, married, one child four years old. Had two miscarriages after birth of baby. Has suffered from backache, bearing-down pains, bladder and bowel troubles, whites, profuse menstruation. When baby was three weeks old, was compelled to be on her feet a great deal, when she commenced to bleed, and continued to lose, often, considerable blood, for two months. She has been treated locally, as well as otherwise, ever since the baby was a year old, and has worn a pessary (large sized Hodge) with much comfort for the two years past. At times she has taken it out, but was compelled to have it reintroduced, to relieve the dragging pains and discomfort she suffered.

Negative galvanism was given, at first fifty and gradually increased to ninety milliampères. Was treated twice a week for eight weeks, and once a week for a month afterward. Sêances eight to ten minutes. Also faradization was given through the vagina by means of a vaginal electrode, and the other, a large, flat clay electrode, was placed on the back. Patient at once improved; in fact every treatment inspired her with confidence, notwithstanding the fact that so many different operations had been made. Moreover, I treated her for months with iodine applications, chloride of zinc, and carbolic acid, and gave her internally pot. iod. and mercury; iron, and muriate of ammonia, in large doses, with but very little permanent benefit. Blisters were also frequently applied externally. Patient was discharged, cured, on August 15, 1889, when I made the following note: Sound enters two and three-quarter inches; womb freely movable; os healthy, and cervical canal free from any thick, glutinous discharge; womb in good position, and vaginal walls much firmer.

I have always supplemented the galvanic treatment in these cases of relaxed vaginæ, with the faradic current—the one electrode in the vagina, and the other over the small of the back—in the hope that it would tone up, not only the vaginal muscular structure, but the womb ligaments, and muscle fibre in them. Moreover, in an extreme case of procidentia uteri, due to great relaxation of the vagina, in fact the procedentia due to the vaginal prolapse, I found the faradic current very serviceable. The patient made the applications herself, night and morning. Previous to the use of the battery, a small-sized Waite and Bartlett, she always wore a good-sized rubber ball in the vagina, to hold the parts in place. She walked with great diffrculty, in fact remained most of the time in her room. In four weeks she dispensed with her support, and found that a napkin between her legs made her sufficiently comfortable, to enable her to get about without any pain, and take such exercise and enjoyment as she had not participated in for years. She continues to take the electricity night aud morning, and says that the dragging pains are felt no more.

CASE III.—*Subinvolution, anteversion, laceration of left cervix, with some eversion of lips, circular erosion around os, endocervicitis and granular degeneration of mucous membrane of body, proved by curette. Sound entered four inches.*

Patient *æt.* 31, married when twenty-two. Menses appeared without much pain and not profuse in amount, at the age of fifteen. Had two children. Always well until after second confinement, when she complained of backache, profuse discharges, and profuse menstruation. Had two miscarriages, one at three months, and the other at seven weeks. Was called to see the patient on May 1, 1889, and found her bleeding profusely. Had been previously quite regular, and this period came on naturally. I prescribed ergot and ice applications over the lower abdominal region.

The bleeding continued so profusely that I deemed it advisable to plug the vagina at my next visit. On the following morning some blood had oozed through the plugs, and the patient was compelled to wear a napkin. These plugs, covered, as they were, with iodoform powder, were left *in situ* three days, when they were carefully removed. The hemorrhage at once returned, and I again plugged the vagina. On the third day these tampons were removed, and the bleeding had apparently ceased, so I did not re-insert the plugs. I was summoned again, post haste, in a few hours, and found her bleeding profusely; large clots of blood filled the vagina, and were seen upon the napkins. In company with Dr. Waite, we gave her a treatment of positive galvanism, seventy-five ma., for ten minutes, which had the effect of completely stopping all hemorrhage. The ergot was discontinued, and no tampons inserted. On the following day the electrode was introduced with some difficulty, and a second treatment given. Three days later another application was made, and then regularly, at intervals. The diagnosis, given above, was made after the hemorrhages had all ceased, and the womb was tolerably firmly contracted.

I continued this treatment for four weeks, using continuously the positive pole, and at the next period, which was also profuse, I made an application to lessen the flow. . In the middle of the month I commenced to use the negative pole, giving seventy-five milliampères of current, and, as I feared, the hemorrhage returned slightly. I then alternated positive and negative galvanic applications until the following period, which was decidedly less in amount, and required no treatment; yet the loss was considerable. I continued this course of treatment, and, with it, vaginal faradization, for two months longer, and found the patient at the next period nearly normal. Four months after treatment was first commenced, by measurement the sound passed in two and one-half inches, and the general condition of the patient was very good. The os was much smaller in size, inversion of the lips had taken place, and, other than a bluish, congested appearance, the cervix looked normal. I continued to give two treatments a month, for two months, and at each sitting used the negative pole, from seventy-five to ninety ma. The last three periods have been normal in amount, and have come on with little or no pain; and at no time has the patient taken to her bed. At the last consultation, in November, I made the following entry in my case book: Menses natural, three napkins a day; no pain; up and at work during the whole period, in fact, ran a sewing machine and made her boy a suit of clothes. The patient has continued under observation, but has required no treatment.

While treating patients with electricity, I have observed that the pains in the ovarian regions and under the ribs very speedily pass away, and particularly if the inactive or external electrode be applied over the painful side. Moreover, the appetite and the condition of the bowels improve. This observation led me to prescribe electricity for constipation, which condition has been relieved, often in the most gratifying way, by faradization as well as galvanism.

For inveterate constipation, I have advised the patient to purchase a small-sized faradic battery, and make the applications night and morning, for a period of at least ten to fifteen minutes. One electrode is placed over the abdomen, and the other over the anal orifice, and as much taken as can be borne. Again, I have advised the large electrode to be placed over the spine, and, with a small sponge electrode the course of the ascending, transverse and descending colon is followed, making deep pressure. Perhaps galvanism acts best. Still the faradic current has done such satisfactory work, and can be taken by the patient herself, that I don't hesitate to recommend it very highly.

You will observe, in these cases I have reported, that I used from fifty to ninety ma. of current; but I don't wish it to be understood that this intensity is always necessary. You will be surprised to see what twenty to thirty ma. of negative galvanism, for six minutes, twice a week, will do for uterine leucorrhea and recent irritative conditions of the canal, and endometrium, when there is little accompanying hyperplasia, and enlargement of the uterine walls. This is no doubt due to the stimulating and alterative character of these weak galvanic currents.

DISCUSSION.

Dr. M. D. Mann said that he was particularly pleased with the use and effect of electricity in the treatment of uterine fibroids; he has treated over thirty of these cases, and, amongst them, large growths, which often seem actually to melt away by the use of the electricity. Dr. Mann has used as many as 200 milliampères, but the patient, as a rule, cannot stand more than 150; he does not believe any one can take a current of 1,000 milliampères, as reported by some observers. He employs Apostoli's method, but used a different external electrode; clay was tried first, and discarded as dirty and cumbersome; the metallic dish-filled-with-water electrode of Martin was also tried, and found wanting.

He now used an electrode made according to Dr. Ford, of Utica; a plate of copper, to the under surface of which is held in place by chamois, applied over a layer of a punk material; this absorbs an enormous amount of water, and is consequently very efficient. A large, flat shot-bag is used to keep the electrode applied to the skin. Internally he at first used platinum, but, finding it so difficult to procure, he used a thick wire of alumnium, covered by a non-conductor to near its end. The negative is, in his practice, used internally, unless there be hemorrhage, when the positive pole is substituted. It may take a few or many sittings to cure a fibroid; they usually diminish ½ to ⅓, but never disappear entirely. This use of electricity is not entirely without danger; in one case he treated a patient for a fibroid, and, after twenty sittings, it had nearly entirely disappeared. After the last application, the patient walked home, and shortly afterward had a chill, followed by a salpingitis which ruptured into the uterus, but which eventuated in recovery; in this case he cannot say exactly what caused the complication.

In another case he had a great slough follow the thrusting of a needle electrode into a fibroid; this case also recovered, but only after having the slough cavity well drained.

In the treatment of dysmenorrhea by electrolysis, he has been both pleased and disappointed; some of the cases benefitted were very gratifying indeed. In the endeavor to absorb by electricity the great pelvic inflammatory products so often met by the gynecologist, he was very greatly disappointed, and he could not get the results in these cases which are claimed in similar cases by others.

Dr. Herman Mynter said that personally he had used electricity in these lines very little, and particularly latterly. He spoke of a case which he had had years ago, before the advent the milliampère metre and other similar instruments of electrical precision, when he had used a high current on a uterine fibroid, and when a severe purulent peritonitis followed.

Dr. Clark asked whether Dr. Hayd had had any experience in the treatment of those cases of scanty menstruation, rather than those of amenorrhea.

Dr. Ingraham said that he had had good results in the treatment of fibroids by electricity, but not at all with its effects on pelvic inflammatory residues. In the treatment of a case of uterine hemorrhage, he had not checked the flow as he expected; why, he could not say.

Dr. S. Y. Howell asked whether any one had good results with electricity in cases of sterility from flexions. In such cases he had often cured the dysmenorrhea, but could not, as yet, state whether the sterility was overcome.

Dr. Mann, partly answering Dr. Howell, mentioned a case of acute retroflexion where a laparatomy was made by a Chicago surgeon, and the uterus attached to the abdominal wall. In this case the cervical and uterine canal was so small as not to admit the smallest probe, and, though repeatedly dilated, always returned to the contracted condition.

After a few applications of a twenty-five milliampère current to the canal, it remained patulous. Whether the patient will now become pregnant or not, he could not say.

Dr. HAYD had (replying to Dr. Clark) had very happy results in cases of dysmenorrhea as well as cases of amenorrhea. He usually uses the negative pole, but if there be much sensitiveness, this is changed for the positive; it is not well, in his judgment, to use seventy-five milliampères at first, but rather employ ten, and longer continued. In the cases of pelvic cellulitis with inflammatory deposits he did not see why these thickening should not disappear before the current, if properly applied, and he believed that they would.

Dr. ROSWELL PARK then read a paper on

### SOME POINTS IN THE SURGERY OF THE ALIMENTARY CANAL.

My remarks to-night are intended to be rather a report of some personal work, with a running commentary thereon, than a formal presentation of anything bearing upon a subject so large that, to treat adequately thereof, would fill a large volume.

Apropos to what has been said to-night of electrolysis, I wish first to report the case of a young woman who had a gastrotomy successfully and skilfully performed last year by Dr. Clark, of Olean, for stricture of the œsophagus. At the time of his operation it was with difficulty that she swallowed fluids. When she came to me in mid-winter, she could not even swallow her saliva; there was dilatation of the œsophagus, above the stricture, with frequent regurgitation of saliva, which had accumulated. I could pass nothing through the stricture, not even fine urethral bougies. It was located about twelve inches from the upper incisors.

I turned out on my lathe some fine olivary tips, which I attached to a coil of wire, inside a rubber tube. With this for a negative pole, the positive being a sponge applied over the epigastrium, and with a current of about 20-25 milliampères, I began treating every other day. After the third treatment she could swallow fluids; after the seventh, she began taking solid food. I can now pass a bougie 5-16 in. in diameter, and she eats everything.

I report this case the more willingly, since it is a brilliant triumph for electrolysis, with which, in the urethra, I have had but little encouragement or success.

During the past eight years I have seen a number of cases of cancer of the stomach, with reference to operative intervention for its relief. Not until last summer did a case which seemed to me suitable present, and then, through the kindness of my friend, Dr. Mann.

I take from my personal records the following history of the case in question:

Mrs. M. J. B., *æt.* 33, married, and has four children. For past five years some indefinite gastric trouble has kept her in poor health. Family history, good. For the past four months she has been conscious of a tumor in the epigastrium, which has grown steadily, and given increasing pain. During the past three weeks she has vomited almost everything she has eaten, and is now much emaciated.

Seen with Dr. Mann.—A distinct and very movable tumor can be felt in the epigastrium, which can be moved upward to the sternum, and downwards to the navel.

Cancer of pylorus diagnosticated, and operation advised. At once put on peptonized milk and tonics.

Operation February 19, 1889—Incision in median line; tumor presented at once, and found to involve pylorus, with quite a portion of the greater gastric curvature. A few mesentric glands involved. Gastro-colic omentum first tied off and separated; then the lesser curvature freed in same way. Next, the stomach divided in such a way as to include about four inches of its greater curvature, and one and a half inches of its lesser; then the duodenum divided. Next, the mucosa of the stomach stitched with interrupted catgut sutures; after this the serosa, and musculosa united with fine silk, down to a point where the duodenum could fit; then the remaining gastric, and the duodenal mucosa united with silk, and over these their serosa and musculosa. Between sixty and seventy silk sutures were used. External wound closed with silver wire. The greatest difficulty was in dealing with hemorrhage from branches of *arteria colica dextra*.

Patient bore operation well, and was put to bed in good condition.

Fed *per rectum* early on the second day, and thereafter.

Everything went favorably till the night of the second day, when she became restless, temperature rose, and she had "sinking" spells.

She grew worse, and died in one of these spells, sixty-three hours after operation.

*Post mortem.*—Stomach found full of blood, which evidently escaped from a vessel in the mucosa, near upper extremity of gastric wound. Not the slightest sign of peritonitis, nor of leakage.

Microscopically.—The tumor was a typical scirrhus, with areas of colloid degeneration.

The specimen which I exhibit shows you that just about one-third of the stomach was removed during the operation. You will see from it, as from the diagrams drawn upon the board, that success in the operative part of the work depends upon strictest attention to detail; that these surfaces and corners must be brought together with scrupulous care, and that, unless serous surfaces are approximated with scrupulous exactness, the union will not be water-tight.

This patient lived over two days, and it will be seen that not the slightest leakage occurred. Death was caused by hemorrhage from a minute vessel in the mucosa, a vessel so small that it would have eluded the observation of any one at the time.

(Diagrams were drawn upon the board to illustrate the technique of the operations upon the stomach, without a reproduction of which a description would be uninteresting.)

With reference to such operations, my calm judgment is that they are feasible and justifiable, but only in well-selected cases.

While not gainsaying its possible advantages in hopeless cases, I cannot deny that it is both a most severe ordeal for the patient, and a trying strain upon the operator.

For cases of cancerous or other stricture anywhere along the intestinal tube, which do not permit of such radical measures, there are yet other methods of affording palliation, if not a prospect of permanent relief. To rehearse all these would be to tax your patience beyond endurance. I prefer to call your attention rather to the new methods of short-circuiting the intestinal canal by means of apposed openings above and below the lesion, the perfection of which are included in what is known as Senn's operations with bone plates, or substitutes thereof. I show you here, for example, the bone plates, as made under Senn's direction; also Abbe's rings of catgut. But the demonstration of some specimens which I have here will better illustrate the advantages of this measure. The first is from a patient who was operated upon a year and a half ago, nearly, but whose case was never reported:

Mrs. B., 47 years of age, was admitted in my service at the Buffalo General Hospital early in February, 1888. She gave the following history: She was the mother of sixteen children, four of whom have died. There was a cancerous family history, though difficult to get at accurately. She was small of stature. She gave an account, on entrance, of some alleged rectal trouble. On examination, I found nothing wrong in the lower bowel, but felt a tumor the size of my clenched fist, in the left hypochondriac region, nearly down in the iliac fossa; the mass being hard, insensitive, movable, and somewhat nodulated. She never complained of nausea, and never vomited; about her only complaints were of constipation and malaise. She said she had noticed the tumor herself for only three or four months. In the past two months she had lost twenty pounds. During her stay in the hospital, this tumor gradually shifted its position, and assumed a position much higher, and nearer the middle line. A careful estimation of urea showed a daily elimination notably below the normal amount. My diagnosis was cancer of the omentum, or of the colon, and, as I watched her for two weeks before the operation, I had no reason to change my opinion as to its location, nor had those of my colleagues who saw her with me. My advice was in favor of exploration and excision, if this were found to be practicable.

February 25, 1888, the operation was undertaken, under chloroform, in my clinic, Prof. Mann kindly assisting me. The patient had been prepared for it in every possible way. The incision was made in the middle line. After opening the peritoneum, the transverse colon presented first, and was seen to be healthy. The tumor was at once searched for, easily found, and, to our surprise, found to involve the stomach, which appeared largely filled up with a firm, nodular growth. The entire pyloric half was too much involved in the growth to permit thought of pylorectomy, so I at once decided to perform the operation described by Dr. Senn at the Washington International Congress. Fortunately I was provided with the decalcified bone plates advised by him, and made under his direction. I made a buttonhole near the cardiac end of the stomach, through which I slipped one plate; then I secured the nearest available loop of small intestine, without reference to determining its exact position, and did the same procedure there. I met with no difficulty in getting the plates into good position, with the vascular walls between them, and then tied them fast. After this the abdominal wound was closed. The patient rallied well from the shock, and, on the following morning, she was in good condition. That afternoon her temperature began to rise; during the evening it rose to nearly 106, and on the morning on the second day, she died.

Complete *post mortem* examination was not permitted by the friends. The external appearance of the wound was good; the abdomen was somewhat distended. On cutting the sutures, it was found that agglutination of wound edges was not perfect, and that the peritoneal edges of the wound gaped. The intestines were distended with gas, and the abdominal cavity was full of a turbid fluid, in which were suspended small shreds and masses of lymph. Fresh lymph was found in numerous places in the intestines, and that noted at the upper part of the abdominal cavity had a decidedly purulent appearance. The site of the operation, however, was in excellent condition. The bone plates, though somewhat softened, were in proper position, and closely approximated; the serous surface inclosed between them were adherent all round, so that no leakage could have taken place; and the artificial opening between the stomach and the small intestines was patulous. Along the greater curvature of the pyloric end of the stomach was found a tumor, irregular in outline, its total mass about the size of a medium-sized orange, and quite soft; no other tumors were detected. Microscopical examination showed this one to be a round-celled carcinoma. Evidently an error had been made in the primary diagnosis, so far as exact site of the tumor was concerned. But she presented none of the ordinary symptoms of dilatation of the stomach; in fact, her food seemed fairly well digested, and she had no nausea or vomiting, and these features, coupled with the fact that the tumor, when first felt by myself, was much nearer the left iliac fossa than the proper position of the pylorus, makes it not surprising that one was misled.

She evidently died of peritonitis; whether this was due to the fact that the laparatomy was made in a public amphitheatre, is not to be easily decided. So far as the success of the measure devised by Dr. Senn is concerned, it was perfectly satisfactory. The viscera were agglutinated so that the opening was water-tight, and, while the case was not a successful one, ample demonstration of the practicability of Senn's operation was furnished.

Reference to this specimen will show that the apposition of serous surfaces was perfect, and union between them rapid and satisfactory. It will be seen that the opening is patulous, although subsequent experience is teaching us all that these openings, as thus made at first, are too small. They will have to be made larger in the future, if they are to be of lasting benefit.

To better illustrate the wide range of applicability of this method, I show you here three specimens from as many dogs. The first is an entero-enterostomy, where small intestine is joined to small intestine. I made this largely to experiment with plates of non-absorbable material, in order to learn whether the operation could not, in an emergency, be done with material that could be quickly prepared. For this purpose

I used plates cut out of medium or large-sized rubber tubing. The needles and catgut sutures are placed as Senn places them. They were inserted into the gut without trouble, fastened, a few Lembert sutures placed around them for security, and when the dog was killed, a few days later, not a trace of them was found. Using such inert material for the plates, I made the sutures of animal tissue, *i. e.*, catgut.

In the second specimen you see an example of a gastro-enterostomy, with perfect results. This animal was not killed for several weeks, and then for another purpose. Then the same rubber plates were used.

In the third specimen I show what may be done in the way of completely removing a section of the alimentary canal, closing the ends by turning them and stitching them snugly, then placing them side by side, and making lateral openings, which shall be then thus utilized by means of plates or rings, so as to preserve the permeability of the intestinal canal. Inasmuch as the gut is now closed at point of section, some absorbable material must be used for the plate or ring. For this instance I imitated Abbe's rings by extemporizing one of the same material, only made with much less care, as one might have to do in an emergency, were he previously unprovided with them. The result is, as you see, again perfect, and this animal was killed some three weeks or so afterward, long enough to amply demonstrate the success of the method.

These preparations by no means illustrate all that can be done in this direction, but I know they will be at least suggestive.

I had thought to-night to detail some successful work in making enterostomy for acute obstruction of the bowels; also some cases of resection of the small intestine, and of the rectum; as well to make some remarks on the benefits of exploratory laparatomy in checking the progress of malignant disease; but the hour is late, and I would defer them to another time rather than tax your patience now.

### DISCUSSION.

Dr. Herman Mynter said that he had had a case in 1876, when he practically used the same method of intestinal short circuiting as Senn's, excepting the bone plates. The case was published, and practically was a case of carcinoma of the intestines where the gut was enormously dilated above, and contracted below, the new growth.

He could not exsect, so he made a button-hole in the intestines above and below the obstruction and sutured the openings together.

The patient's bowels afterward moved, the first time in many days, but he died of exhaustion shortly after the operation.

Dr. Wm. C. Phelps was present at the operation, related by Dr.

Park, for the relief of pyloric cancer by exsection, and could corroberate what he had said about the apparent freedom from hemorrhage after the gastric wall had been sutured.

He did not see any way to have avoided the hemorrhage, and thought that this might be a point to count against the method employed.

Dr. ROCHESTER drew attention to the difficulty often encountered in trying to localize an abdominal growth, and mentioned a case when before death there seemed to be but a small lump in the abdomen, while the post mortem revealed extensive invasion of the viscera by carcinoma.

Dr. PARK, in closing, drew attention to the difference in the size of the openings in Senn's plates and Abbe's rings. The openings in the plates certainly are small, and six months after operation the corresponding intestinal openings would probably have contracted very materially.

Dr. JONES reported a case of pyonephrosis, and presented the specimen from the same.

The following resolutions were unanimously adopted:

*Resolved:* That we receive with feelings of profound regret the sad intelligence of the death of Dr. Phineas H. Strong, one of the Association's oldest, most zealous, and most regular members.

*Resolved:* That we extend to the family our most heartfelt sympathy in this their hour of sore bereavement; and trust their grief may be lessened by the remembrance of his good work and many excellent traits.

*Resolved:* That copies of these resolutions be sent to the family, and to the BUFFALO MEDICAL AND SURGICAL JOURNAL.

Signed, J. B. SAMO,
J. B. COAKLEY,
HERMAN E. HAYD.

The next meeting (April 1st) was announced as the "annual meeting," when the election of officers would ensue, and the programme for the succeeding six months would be made up.

---

The annual meeting was held Tuesday evening, April 1st, in the Library of the Polytechnic Institute, No. 9 West Mohawk street, with the President, Dr. A. A. HUBBELL, in the chair.

Drs. B. N. Strong, J. W. Putnam, John Parmenter, and W. H. Heath were elected to membership.

The names of Drs. M. A. Crockett and A. L. Benedict were proposed for membership.

The following officers for the ensuing year were then elected:

*President*—DR. A. A. HUBBELL.
*Vice-President*—DR. W. C. PHELPS.
*Secretary*—DR. W. H. BERGTOLD.
*Treasurer*—DR. F. E. L. BRECHT.
*Librarian*—DR. W. H. HEATH.

